# Supplementary material for: Pluripotent Cells Expressing APOE4 Exhibit a Pronounced Pro-Apoptotic Phenotype Accompanied by Markers of Hyperinflammation and a Blunted NF-κB Response
Source: Int J Mol Sci. 2025 Sep 23;26(19):9283. doi: 10.3390/ijms26199283 (PMC12524803; doi:10.3390/ijms26199283)
Supplement: Supplementary file 1 [file ijms-26-09283-s001.zip › ijms-3849513-supplementary.pdf]

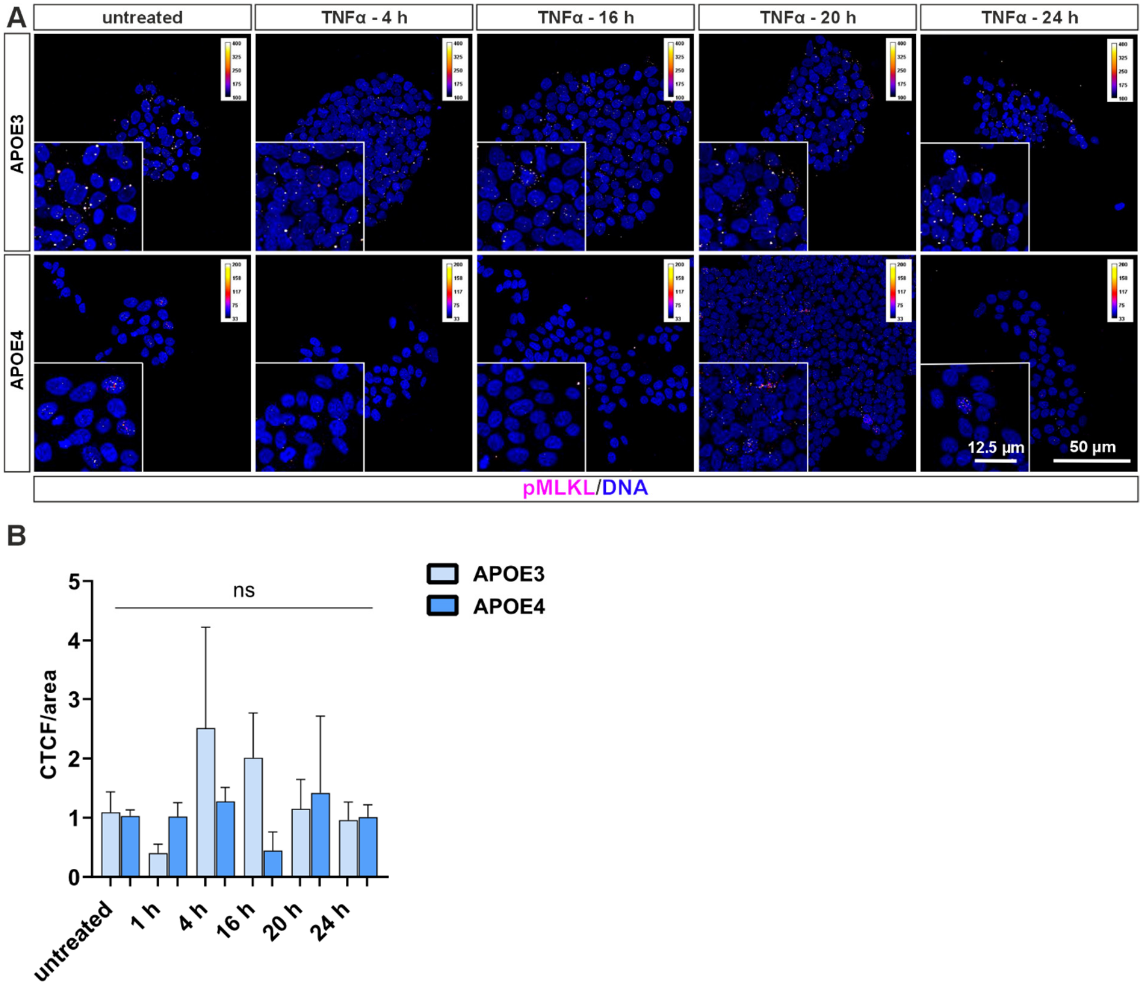

Figure S1: Analysis of the cell death mechanism necroptosis in APOE-expressing cells. (**A**) Immunocytochemical staining and false color analysis for phosphorylated MLKL (pMLKL), a necroptosis marker, revealed expression in both APOE3 and APOE4 cells following TNF-α treatment. (**B**) Quantification of corrected total cell fluorescence (CTCF) showed no significant difference in pMLKL levels between the two cell lines. Statistical analysis was performed using one-way ANOVA (n = 3), followed by multiple comparisons corrected using the False Discovery Rate (FDR) method of Benjamini, Krieger, and Yekutieli. *$q \leq 0.05$, **$q \leq 0.01$, ***$q \leq 0.001$, ****$q \leq 0.0001$. Data are presented as mean ± standard error of the mean.
